# Assessment of Thyroid Status and Six Months of Follow-Up in COVID-19 Patients: A Prospective, Observational Study

**DOI:** 10.7759/cureus.81896

**Published:** 2025-04-08

**Authors:** Hasan M Rashed, Mohammad Monzurul Alam Bhuiyan, Md. Abdul Matin, Abed H Khan, Shohael Mahmud Arafat

**Affiliations:** 1 Internal Medicine, Bangabandhu Sheikh Mujib Medical University, Dhaka, BGD; 2 Laboratory Medicine, Bangabandhu Sheikh Mujib Medical University, Dhaka, BGD

**Keywords:** bangladesh, coronavirus disease 2019, covid thyroiditis, post-covid sequelae, sars-cov-2, thyroid status, viral thyroiditis

## Abstract

Background

Globally, the COVID-19 pandemic inflicted considerable morbidity and mortality. It has become a systemic illness that affects several body organs. The primary pathway for the SARS-CoV-2 virus to enter cells is the angiotensin-converting enzyme 2 (ACE2) receptor, and it uses transmembrane serine protease 2 (TMPRSS2) for S protein priming. Thyroid follicular cells have been found to express ACE2 and TMPRSS2 at even higher levels than those found in the lungs. The objective of the study was to assess and determine the six-month clinical outcome of thyroid dysfunction in COVID-19 patients.

Methods

This prospective, observational study was carried out in the COVID-19 unit of Bangabandhu Sheikh Mujib Medical University, Dhaka, Bangladesh. Forty-eight hospital-admitted COVID-19 reverse transcription-polymerase chain reaction (RT-PCR)-positive patients were included as per inclusion and exclusion criteria, and there was no control group. All admitted patients were classified into non-severe (mild, moderate) and severe (severe and critical) disease as per national guidelines. Baseline laboratory tests for thyroid-stimulating hormone (TSH), free T3, and free T4 were done on admission. The patients were again categorized according to their thyroid status. Further evaluation was done by performing autoantibody tests (TSH receptor Ab, anti-thyroid peroxidase Ab, anti-thyroglobulin Ab). The patients were followed up in the first month and sixth month to see disease progression and outcomes.

Results

Age and comorbidities were associated with an increased risk of severe disease. A total of 35 (73%) patients had comorbidities, 23 (49%) had multiple comorbidities, and 12 (24%) had single comorbidities. All patients (n=48) presented with fever and cough; 45 (94%) patients had fatigue, palpitations were present in 35 (73%) patients, and dyspnea in 32 (67%) patients. The difference in mean values of TSH, FT4, and FT3 among severe and non-severe groups was found to be significant. Most of the patients (n=36; 75%) were in a euthyroid state at baseline. Among the rest, 25% had thyroid dysfunction, including one (2.08%) who had subacute thyroiditis and eight (16.67%) who had euthyroid sick syndrome, which was higher in the severe group (14.59%). Three (6.24%) patients were autoantibody-positive and were diagnosed as having Hashimoto's thyroiditis in the form of primary hypothyroidism (2.08%; n=1) and subclinical hypothyroidism (4.16%; n=2). The Spearman correlation coefficient showed a strong negative correlation between severity and TSH (rs = -0.71) and moderate positive correlations between severity and free T3 (rs = 0.63) and free T4 levels (rs = 0.53). Follow-up at the first month showed that 44 (91.96%) returned to a euthyroid state, and at six months, 45 (93.7%) returned to a euthyroid state.

Conclusion

Thyroid hormone levels appear to change and recover gradually and spontaneously. Most patients have normal thyroid function status during COVID-19 infections, but a considerable number of patients can develop euthyroid sick syndrome in severe COVID-19 infections, from which they recover spontaneously.

## Introduction

The endocrine system remains potentially susceptible to damage by COVID-19 primarily because of the high expression of angiotensin-converting enzyme 2 (ACE2) receptors, mainly in the pituitary, pancreas, and adrenal glands, and testes [1-3]. The thyroid is one of several endocrine organs that have been demonstrated to be impacted by the SARS-CoV-2 virus. Various changes in thyroid function have been noted during and after COVID-19 infection; some of these changes are temporary, while others are linked to long-term consequences [4,5]. One type of granulomatous thyroiditis that can develop during or following a viral disease is subacute thyroiditis (SAT) [6]. Numerous case reports of thyroiditis linked to COVID-19 have been released, and several possible pathophysiological explanations have been put up. Thyroid follicular cells have been found to express ACE2 and transmembrane serine protease 2 (TMPRSS2), which act as entry points for SARS-CoV-2, at even higher levels than those found in the lungs. However, more research is necessary to determine their precise pathogenetic role in post-COVID-19 thyroiditis [7]. 

Following COVID-19 infection, thyroiditis has been documented during both the active phase and the post-viral follow-up phase. The early reports of COVID-19-associated SAT suggested a similar presentation to other post-viral SAT aetiologies, with neck pain being a prominent characteristic [8-10]. According to Muller et al., there are no local symptoms, and the presentation is unusually "painless" [11]. Significant neck pain was a common presentation in the few COVID-19-related thyroiditis cases that were reported from different parts of the world, despite variations in other clinical and biochemical characteristics [12,13].

The effect of COVID-19 on thyroid function is currently a topic of growing investigation. So, it is essential to evaluate thyroid function status in our setting. To assess the thyroid function status, COVID-19 patients were investigated and followed up to find out whether this abnormality was transient or permanent.

## Materials and methods

This was a prospective, longitudinal study conducted at the Department of Internal Medicine, Bangabandhu Sheikh Mujib Medical University (BSMMU), Dhaka, Bangladesh, from August 2021 to May 2022. The study was approved by the Institutional Review Board, BSMMU (approval number: BSMMU/2022/5934). The nature and goal of the study were thoroughly explained to each participant. Prior to participation, each patient provided written consent that was understood and informed. Every patient had the freedom to take part in, decline, and leave the study at any time during its duration.

Participants

Adult patients (≥ 18 years) who were COVID-19-positive according to the reverse transcriptase polymerase chain reaction (RT-PCR) test and were willing to take part in the study were included. We excluded patients with a history of thyroid dysfunction and those taking medications that affect thyroid function (such as amiodarone, lithium, and oral contraceptives).

Patients were recruited from triage and the COVID-19 unit at BSMMU for the study, and further follow-up was done at follow-up visits at the post-COVID outpatient clinic at BSMMU. We divided the patients into two groups: (i) the non-severe group, which consisted of mild COVID and moderate COVID patients, and (ii) the severe group, which consisted of severe COVID and critical COVID patients. Mild, moderate, severe, and critical COVID-19 were classified according to the World Health Organisation's (WHO) severity classification [14], as follows: (i) Critical COVID-19: defined by the criteria for acute respiratory distress syndrome (ARDS), sepsis, septic shock, or other conditions that would normally require the provision of life-sustaining therapies such as mechanical ventilation (invasive or non-invasive) or vasopressor therapy, (ii) Severe COVID-19: defined by any of the following: oxygen saturation < 90% on room air; signs of severe respiratory distress (accessory muscle use, inability to complete full sentences, or respiratory rate > 30 breaths per minute), (iii) Moderate COVID-19: fever and respiratory symptoms with radiological findings of pneumonia, respiratory distress with <30 breaths per minute, and pulse oximetry showing saturation > 93% at ambient air, and (iv) Mild COVID-19: clinical symptoms are fever, cough, sore throat, malaise, headache, muscle pain without shortness of breath, or abnormal imaging.

Sample size calculation

Details of the sample size calculation are given as follows [15]:

$n = \frac{(Z_{a/2}+Z_{\beta})^{2}\times (\pi_{1}(1-\pi_{1})+\pi_{2}(1-\pi_{2}))}{(\pi_{1}-\pi_{2})^{2}}$

in which, n = required sample size per group

π₁ = first proportion (e.g., 0.15)

π₂ = second proportion (e.g., 0.02)

Zₐ/₂ = Z value for desired significance level (e.g., 1.96 for 5%)

Zᵦ = Z value for desired power (e.g., 0.84 for 80%)

so that $n = \frac{(1.96 + 0.84)^{2}\times (0.15 &times; 0.85 + 0.02 &times; 0.98)}{(0.13)^{2}}$

n = 23.5 for each group. So, we enrolled 24 in each group, 48 participants in total.

Study procedure

Proper history and physical examination were done, including pulse, blood pressure, oxygen saturation, weight measurement, and chest examination, and the patient's blood was collected. Each participant went through two follow-ups, scheduled in the first month and the sixth month from the date of the first blood sample testing for thyroid function test. We recruited a total of 48 participants, 24 in each severe and non-severe group.

Thyroid Function and Autoantibodies

Using venipuncture, blood samples were first obtained for serum thyroid-stimulating hormone (TSH), free triiodothyronine (FT3), and free thyroxine (FT4) in ethylenediaminetetraacetic acid (EDTA) and plain tubes. If the TSH was low along with high FT3 and FT4, we performed an anti-thyroid receptor antibody (TRAb); if only TSH was low and FT4 and FT3 were normal, then radioiodine uptake tests were performed. For patients with high TSH with or without FT3 and FT4 abnormality, anti-thyroperoxidase (anti-TPO) and anti-thyroglobulin Ab (anti-TG) tests were performed. For TSH, FT3, and FT4, the automated analyzer Atellica® (Siemens Healthineers AG, Erlangen, Germany), was used, and thyrotropin receptor antibody (TRAb), anti-thyroid peroxidase (TPO), and anti-thyroglobulin antibody (anti-TgAb) were done by using chemiluminescent methods on the DxI 800 Access® immunoassay system (Beckman Coulter, Inc., Brea, California, United States). TRAb was determined using time-resolved amplified cryptate emission (TRACE) on the Kryptor analyzer (Thermo Fisher Scientific Inc., Waltham, Massachusetts, United States).

Statistical analysis

The statistical analysis was conducted using IBM SPSS Statistics for Windows, Version 26.0 (Released 2019; IBM Corp., Armonk, New York, United States). Age, TSH, FT3, FT4, and other continuous variables were displayed as mean ± SD and median ± interquartile range (IQR). Frequency and percentages were used to represent categorical factors, such as gender. A p-value of less than 0.05% was deemed significant, and a significance criterion of 5% was established. The qualitative and quantitative data were analyzed using the chi-square test and the unpaired t-test. Group comparisons were conducted using a one-way ANOVA.

## Results

Baseline characteristics

Of the 48 patients who were enrolled in the trial, the non-severe group consisted of 24 (50%) patients, and in the severe group, there were 24 (50%) patients. In the non-severe group, 20 had moderate severity, and four had mild severity. The remaining 24 patients were in the severe group; among them, 19 were severe, and five were critical, requiring ICU admission. All patients who enrolled went through two follow-ups.

**Figure 1:**
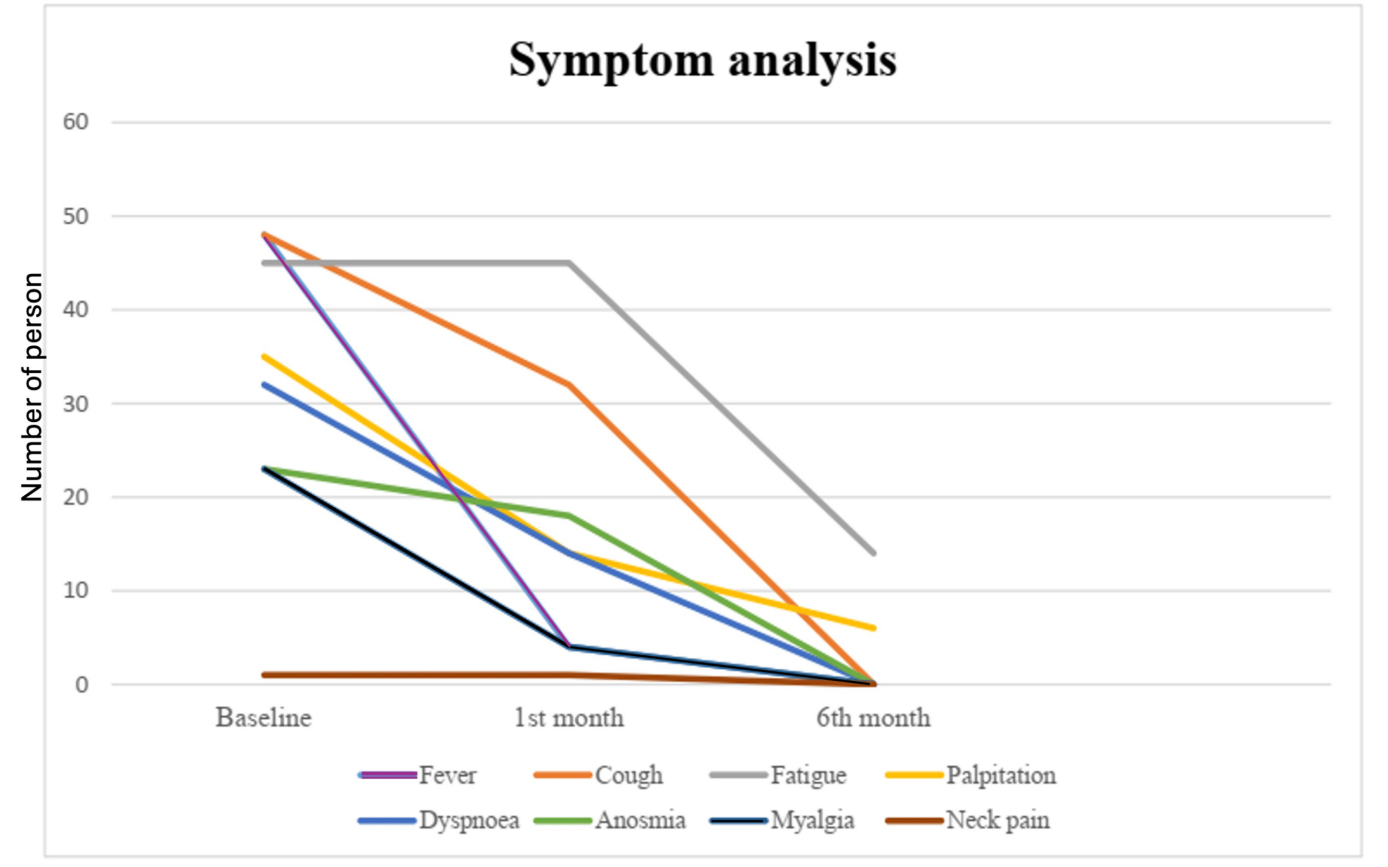
Primary presentations of COVID-19 patients during their enrollment and follow-up

The majority of patients were male (62.5%, 30), and the male:female ratio was 15:9. Of the participants, 25 (52%) were more than 60 years old. Among the patients with severe diseases (n = 24), 16 (66.6%) patients were more than 60 years old (p 0.04). Thirty-five (73%) patients had comorbidities, and among them, 23 (48%) had multiple comorbidities, which is a significant association with severe disease (p < 0.05). Of the patients, 58% had hypertension, 52% had diabetes, 17% had coronary artery disease, 15% had asthma, 12% had chronic obstructive pulmonary disease, 8% had chronic kidney disease, and 2% had cerebrovascular disease. The body mass index (BMI) for non-severe patients was 23.9 (21.9-25.8) kg/m^2^, and for severe patients, it was 24.7 (23.3-28.6) kg/m^2^ (p = 0.16) (Table 1). Figure 1 shows the baseline symptoms and symptoms complained of by participants during the first and second follow-ups.

**Table 1:** Baseline characteristics of the patients BA: bronchial asthma, CKD: chronic kidney disease, CLD: chronic liver disease, CPD: chronic pulmonary disease, COPD: chronic obstructive pulmonary disease, CAD: coronary artery disease, CVD: cerebrovascular disease * Mann-Whitney U test was done, **Chi square test was done

Characteristics	Total (n=48)	Non severe (n=24)	Severe (n=24)	Chi square value	P value
Age category (years), median+IQR	61 (47-65.5)	52.5 (43.5-62)	65 (58-70)		0.024*
Age category (years), n (%)					
< 60	23 (47.9%)	15 (62.5%)	8 (33.3%)	2.6	0.04**
>60	25 (52.1%)	9 (37.5%)	16 (66.6%)		
Sex, n (%)					
Male	30 (62.5%)	16 (66.6%)	14 (58.3%)	2.8	0.031**
Female	18 (37.5%)	8 (33.3%)	10 (41.6%)		
BMI, mean (range)	24.2	23.9 (21.9-25.8)	24.7 (23.8-28.6)	0.9	0.16**
Comorbidities (Yes), n (%)					
Diabetes mellitus	25 (52%)	6 (25%)	19 (79.1%)	4.2	0.0003**
Hypertension	28 (58%)	10 (41.6%)	18 (75%)	3.1	0.01**
CAD	8 (17%)	2 (8.3%)	6 (25%)	2.1	0.06**
Asthma	7 (15%)	2 (8.3%)	5 (20.8%)	1.9	0.08**
COPD	6 (12%)	1 (4.1%)	5 (20.8%)	3.2	0.01**
CVD	1 (2%)	0	1 (4.1%)	1.7	0.08**
Obesity	15 (31%)	8 (33.3%)	7 (29.1%)	1.1	0.12**
CKD	4 (8%)	0	4 (16.6%)	2.6	0.04**

Regarding TSH levels, in the non-severe group, one (4.17%) had elevated, one (4.17%) had low, and 22 (91.66%) patients had normal TSH levels. In contrast, in the severe group, two (8.33%) had elevated, eight (33.33%) had low, and 14 (58.34%) had normal TSH levels (p < 0.05). Regarding FT4 levels, in the non-severe group, all the patients had normal FT4 levels. In contrast, in the severe group, three (12.5%) had elevated, one (4.17%) had low, and 20 (88.33%) had normal FT4 levels (p < 0.05). In the case of FT3 level, in the non-severe group, all the patients had normal FT3 levels. In contrast, in the severe group, one (4.17%) had elevated, one (4.17%) had low, and** **22 (91.66%) had normal FT3 levels(p > 0.05) (Table 2).

**Table 2:** Baseline TSH, FT3, FT4 in the severe and non-severe patients TSH: thyroid-stimulating hormone; FT3: free T3; FT4: free T4 Chi-square test was done

Months	Status	Non-severe (n-24), n (%)	Severe (n=24), n (%)	Chi square value	p value
	Elevated	1 (4.17%)	2 (8.33%)	7.56	0.02
TSH	Normal	22 (91.66%)	14 (58.34%)		
	Low	1 (4.17%)	8 (33.3%)		
	Elevated	0	3 (12.5%)	4.36	0.11
FT4	Normal	24 (100%)	20 (83.3%)		
	Low	0	1 (4.17%)		
	Elevated	0	1 (4.17%)	2.09	0.34
FT3	Normal	24 (100%)	22 (91.6%)		
	Low	0	1 (4.17%)		

Table 3 describes the correlation coefficient between severity and TSH (rs = -0.71), between severity and FT4 (rs = 0.53), and between severity and FT3 (rs = 0.63). This result signifies that between severity and TSH, there is a strong negative correlation, and between severity and FT3 and FT4, there is a moderate positive correlation.

**Table 3:** Correlation between disease severity and baseline TSH, FT3, FT4 TSH: thyroid-stimulating hormone Spearman’s Rank Correlation Coefficient Test was done

			TSH	FT4	FT3
Severity		Correlation coefficient (rs)	-0.71	0.53	0.63
		Sig. (2-tailed)	0.05	0.019	0.023

**Figure 2:**
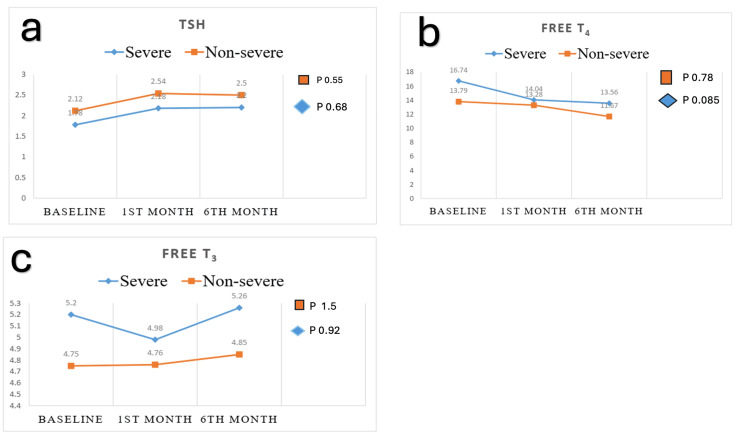
Comparison of TSH, FT4, FT3 between severe and non-severe group at baseline, first month, and sixth month a) Comparison of TSH at baseline, 1st month, and 6th month in the non-severe (p-value 0.55) and severe groups (p-value 0.68). b) Comparison of FT4 at baseline, 1st month, and 6th month in the non-severe (p-value 0.78) and severe groups (p-value 0.085). c) Comparison of FT3 at baseline, 1st month, and 6th month in the non-severe (p-value 1.5) and severe groups (p-value 0.92). Value shown for FT4 and FT3 in pmol/L and for TSH in mIU/ml Repeated measure one-way ANOVA was applied TSH: thyroid-stimulating hormone; FT3: free triiodothyronine; FT4: free thyroxine

Figure 2 shows the trend of TSH levels, which rose in the first month and then became static in both the non-severe and severe groups. In the non-severe group, the mean TSH (uIU/mL) at the baseline was 2.12±1.3; at the first-month follow-up, it was 2.54±1.15, and at the sixth-month follow-up, it was 2.5±1.75. In the severe group, the baseline was 1.78±2.1; in the first month, it was 2.18±1.59, and in the sixth month, it was 2.2±1.59.

In the case of FT4 (pmol/l), a similar trend of falling was observed in the first and sixth months of follow-up in both the non-severe and severe groups. In the non-severe group, the mean FT4 (pmol/l) at the baseline was 13.79±1.97, in the first month 13.28±1.49, and in the sixth month of follow-up, it was 11.67±1.49. In the severe group, the baseline was 16.74±4.05, at the first month, it was 14.04±2.89, and at the sixth month, it was 13.56±2.66. However, in the case of the FT3 level, it didn’t follow a similar trend like in TSH and FT4.In the non-severe group, the mean FT3 (pmol/l) at the baseline was 4.75±0.96, at the first month it was 4.76±0.82, and at the sixth month it was 4.85±0.84. In the severe group, the baseline was 5.2±0.38, at the first month it was 4.98±0.06, and at the sixth month it was 5.26±0.64.

Among 48 patients, there were 36 (75%) in a euthyroid state, six (12.5%) in a subclinical thyrotoxicosis state, two (4.17%) in a sick euthyroid/sub-acute thyroiditis state, one (2.08%) in a thyrotoxic state, two (4.17%) in a subclinical hypothyroid state, and one (2.08%) in a hypothyroid state. 

In total, we found 12 patients with thyroid dysfunctions, with a frequency of 25% at baseline. Among these 12 patients, nine had low TSH. Of these nine patients, only three patients had FT4 and FT3 abnormalities for which we performed TRAb, and others with normal FT4 and FT3 levels for which we performed the radioiodine uptake test. Radioiodine uptake showed one patient with low uptake and diagnosed as a case of subacute thyroiditis, and eight patients were diagnosed as sick euthyroid due to normal uptake.

All three patients with high baseline TSH were autoantibody positive and diagnosed as having Hashimoto’s thyroiditis. Based on low FT4 and FT3, one patient was diagnosed with primary hypothyroidism; the other two patients had normal FT4 and FT3 levels and were diagnosed with subclinical hypothyroidism.

Table 4 shows a total of 36 (75%) patients were euthyroid; the most common thyroid dysfunction we observed was euthyroid sick syndrome (ESS) in eight (16.67%) patients. Most of the patients with ESS were in the severe group, seven (29.2%) in number, and one (4.1%) in the non-severe group. Only one (2.08%) patient in the severe group developed subacute thyroiditis. Follow-up at the first month shows that most of the patients, 44 (91.96%), returned to a euthyroid state; one (2.27%) remained in a sub-acute thyrotoxicosis state, and three (6.81%) were diagnosed as having Hashimoto’s thyroiditis. Among the three Hashimoto’s thyroiditis patients, two patients were in a subclinical hypothyroid state, and one patient was in a primary hypothyroidism state. At the sixth month, most of the patients, 45 (93.7%), returned to a euthyroid state; two patients were in a subclinical hypothyroid state, and one patient was in a primary hypothyroid state as Hashimoto’s thyroiditis. The two patients with subclinical hypothyroidism were not put on thyroxine replacement, but the one patient with primary hypothyroidism was on thyroxine replacement.

**Table 4:** Distribution of thyroid status at baseline, first month follow-up, and sixth month follow-up Chi-square test was done

Timeline	Thyroid status	Total (n=48), n (%)	Non-severe (n=24), n (%)	Severe (n=24), n (%)	Chi square value	p value
Baseline	Euthyroid	36 (75%)	22 (91.6%)	14 (58.3%)	5.44	0.020
	Sub-acute thyroiditis	1 (2.08%)	0	1 (4.1%)		
	Sick euthyroid	8 (16.67%)	1 (4.1%)	7 (29.2%)		
	Hashimoto’s thyroiditis	3 (6.25%)				
	Subclinical hypothyroidism (Hashimoto’s)	2 (4.17%)	1 (4.1%)	1 (4.1%)		
	Primary hypothyroidism (Hashimoto’s)	1 (2.08%)	0	1 (4.1%)		
1^st^ month	Euthyroid	44 (91.6%)	23 (95.8%)	21 (87.5%)	0.27	0.602
	Sub-acute thyroiditis	1 (2.27%)	0	1 (4.1%)		
	Sick euthyroid	0				
	Hashimoto’s thyroiditis	3 (6.81%)				
	Subclinical hypothyroidism (Hashimoto’s)	2 (4.54%)	1 (4.1%)	1 (4.1%)		
	Primary hypothyroidism (Hashimoto’s)	1 (2.27%)	0	1 (4.1%)		
6^th^ month	Euthyroid	45 (93.7%)	23 (95.8%)	22 (91.6%)	0.00	1
	Sub-acute thyroiditis	0				
	Sick euthyroid	0				
	Hashimoto’s thyroiditis	3 (6.3%)				
	Subclinical hypothyroidism (Hashimoto’s)	2 (4.1%)	1 (4.1%)	1 (4.1%)		
	Primary hypothyroidism (Hashimoto's)	1 (2%)	0	1 (4.1%)		

## Discussion

In this study, we aimed to explore the thyroid function status of COVID-19 patients with severity. This prospective study assessed the six-month outcomes of thyroid function status. Basic sociodemographic characteristics were also reviewed. We enrolled 48 COVID-19 patients; of them, four patients had mild COVID-19, 20 patients had moderate COVID-19, 19 patients had severe COVID-19, and five patients were critical. The cohort had 62.5% male patients and 37.5% female, a ratio of 15:9. There was no significant gender difference among different severities (p > 0.05), which is similar to a previous study [16]. The median+IQR of age of all patients was 61 (47-65.5); among them, for the non-severe group, it was 52.5 (43.5-62), and for the severe group, it was 65 (58-70). There is a significant difference in the median age of patients among different severities (p < 0.05), which is similar to previous studies [17]. 

In our study, we found that nine patients (18.75%) had TSH levels below the reference range, whereas the remaining 36 (75%) and three (6.25%) had normal and high TSH, respectively. The results align with the earlier research. According to the THYROCOV study, 20.2%, 5.2%, and 74.6% of patients had low TSH, high TSH, and normal levels, respectively [18]. Low TSH is the most common thyroid issue in COVID-19 patients, which can be attributed to different underlying causes. Proinflammatory cytokine IL-6 can cause negative associations with TSH, resulting in its suppression. Direct follicular damage of the thyroid also occurred due to SARS-CoV-2, resulting in low THS. Another cause might be the presence of cortisol, even in physiological amounts, which can cause suppression of TSH [2,19]. This syndrome is caused by several causes, such as changes in peripheral thyroid hormone uptake, discrepancies in TSH secretion, and modulation of thyroid hormone binding to transport proteins. The pathologic reaction to acute sickness is this disorder [4]. The most typical alterations are normal or low T3, raised T4, and low serum TSH [20,21]. Thyroid dysfunction was shown to be more common in severe COVID-19 patients, with a statistically significant difference (p < 0.005), than in the non-severe group. Specifically, severe COVID-19 patients had statistically significantly lower mean TSH readings than the non-severe group, which is nearly consistent with numerous other research [11,19]. There was a correlation between the severity of the condition and the extent of the decline in TSH and FT3 and FT4 levels. As with many other studies, there was a positive correlation between disease severity and the levels of TSH decline and both FT3 and FT4 increase [22-24].

In our initial assessment, we found that most of the patients were in a euthyroid state; 36 (75%) patients remained euthyroid, and the remaining 12 (25%) patients had thyroid dysfunction. Two (4.17%) of them were in a sick euthyroid state, and six (12.5%) were in a subclinical thyrotoxicosis state. Sick euthyroid is a condition where FT3 is low, and TSH is normal, FT4 may or may not be lower in amount [24]. According to Giovanella et al., the majority of COVID-19 patients had normal TSH values (44-94%) and were euthyroid. In studies on individuals with COVID-19, the frequency of thyroid dysfunction varies significantly, from 13% to 64% [25]. Similar to our study, a positive connection between thyroid malfunction and the clinical severity of COVID-19 was described by Burekovic et al. [26]. To address the low TSH level of nine patients, we perform TRAb and radioiodine uptake tests following the discharge of these patients. Only one patient showed low uptake on the radioiodine uptake test, but the remaining eight patients had normal results. All nine patients were TRAb negative. We concluded the diagnosis as subacute thyroiditis of one (2.08%) patient in the severe group. Among the eight (16.67%) patients, seven (14.59%) in the severe group and one (2.08%) in the non-severe group were diagnosed as having ESS. Zou et al. showed that 27.52% of COVID-19 patients in their study developed ESS, which is also related to the disease severity of patients with COVID-19. After completing the sixth-month follow-up, we found that most of the patients (n=45, 93.7%) had returned to a euthyroid state. Khoo et al. had shown a similar pattern of results [21], which is more consistent with our study. Finally, our study also showed that SARS-CoV-2-induced subacute thyroiditis and ESS were usually reversible, which is supported by a previous study [26].

Limitations of the study

The study has several limitations. First, this study was conducted in a single centre with a small sample size, which is not representative of our whole population. We didn’t have the prior thyroid function status for the study populations, which might raise a question about the preexisting hidden thyroid dysfunction. Our study did not include a control group, which limits our ability to differentiate COVID-19-induced thyroid dysfunction from normal population-level thyroid variations. Additionally, the variable interval between symptom onset and hospital admission may have influenced thyroid hormone levels, potentially affecting our assessment of the temporal progression of thyroid abnormalities. The lack of follow-up antibody testing of the autoantibody-positive patients was another limitation as the persistence or disappearance of autoantibody may have interfered with the outcome. A complete diagnosis of thyroid dysfunctions could not be made due to resource limitations. Thyroid scans, ultrasounds of the thyroid gland, and other related investigations could not be done. We performed a correlation analysis, but a logistic regression analysis might help with adjusting the confounders. Additionally, studies with a larger population with a longer follow-up might validate the findings in our study and provide a deeper understanding of the dynamics of COVID-19 and thyroid dysfunction.

## Conclusions

Thyroid hormone levels appear to change and recover gradually and spontaneously. Most of the patients have normal thyroid function status during COVID-19 infections, but a considerable number of patients can develop ESS, and a small number of patients can develop subacute thyroiditis, and these abnormalities may appear more in severe COVID-19 infection. These types of thyroid dysfunction recovered spontaneously and needed no intervention, but future follow-up of these cohorts is needed to see any change in the pattern. 
